# Load-Induced Glenohumeral Translation After Rotator Cuff Tears: Protocol for an In Vivo Study

**DOI:** 10.2196/43769

**Published:** 2022-12-23

**Authors:** Eleonora Croci, Franziska Eckers, Corina Nüesch, Soheila Aghlmandi, Balazs Krisztian Kovacs, Jeremy Genter, Daniel Baumgartner, Andreas Marc Müller, Annegret Mündermann

**Affiliations:** 1 Department of Orthopaedics and Traumatology University Hospital Basel Basel Switzerland; 2 Department of Biomedical Engineering University of Basel Basel Switzerland; 3 Department of Clinical Research University of Basel Basel Switzerland; 4 Department of Spine Surgery University Hospital Basel Basel Switzerland; 5 Meta-Research Centre Department of Clinical Research University Hospital Basel Basel Switzerland; 6 Department of Radiology University Hospital Basel Basel Switzerland; 7 Institute of Mechanical Systems Zurich University of Applied Sciences Winterthur Switzerland

**Keywords:** abduction, shoulder, rotator cuff, humeral head migration, fluoroscopy, MRI, motion capture, dynamometer

## Abstract

**Background:**

Rotator cuff tears are a common shoulder injury, but they sometimes remain undiagnosed, as symptoms can be limited. Altered shoulder biomechanics can lead to secondary damage and degeneration. In biomechanical analyses, the shoulder (ie, the glenohumeral joint) is normally idealized as a ball-and-socket joint, even though a translation is often observed clinically. To date, no conclusive changes in glenohumeral translation have been reported in patients with rotator cuff tears, and it is unknown how an additional handheld weight that is comparable to those used during daily activities will affect glenohumeral translations in patients with rotator cuff tears.

**Objective:**

This study aims to assess the load-induced glenohumeral translation (liTr) in patients with rotator cuff tears and its association with the load-induced changes in muscle activation (liMA).

**Methods:**

Patients and asymptomatic controls will be recruited. Participants will fill out health questionnaires and perform 30° arm abduction and adduction trials, during which they will hold different handheld weights of a maximum of 4 kg while motion capture and electromyographic data are collected. In addition, fluoroscopic images of the shoulders will be taken for the same movements. Isometric shoulder muscle strength for abduction and rotation will be assessed with a dynamometer. Finally, shoulder magnetic resonance images will be acquired to assess muscle status and injury presence. The dose-response relationship between additional weight, liTr, and liMA will be evaluated.

**Results:**

Recruitment and data collection began in May 2021, and they will last until the recruitment target is achieved. Data collection is expected to be completed by the end of 2022. As of November 2022, data processing and analysis are in progress, and the first results are expected to be submitted for publication in 2023.

**Conclusions:**

This study will aid our understanding of biological variations in liTr, the influence of disease pathology on liTr, the potential compensation of rotator cuff tears by muscle activation and size, and the association between liTr and patient outcomes. The outcomes will be relevant for diagnosis, treatment, and rehabilitation planning in patients with rotator cuff tears.

**Trial Registration:**

ClinicalTrials.gov NCT04819724; https://clinicaltrials.gov/ct2/show/NCT04819724

**International Registered Report Identifier (IRRID):**

DERR1-10.2196/43769

## Introduction

The shoulder (ie, glenohumeral joint) is a unique joint primarily stabilized by the rotator cuff muscles [1]. These muscles facilitate shoulder motion and center the glenohumeral joint [2]. This type of stabilization facilitates a large range of motion [2], which is a prerequisite for many daily, occupational, and recreational activities. An injury to the rotator cuff can therefore lead to an unstable joint and affect joint functionality, negatively affecting patients’ activities and quality of life. Furthermore, altered shoulder biomechanics can lead to secondary damage and degeneration, such as tendinopathy or osteoarthritis [3]. The motion of a healthy shoulder mainly comprises rotation and very small to no translation due to stabilization through muscle forces. Glenohumeral stability is affected by the muscle cross-sectional area; the level of muscle activation; and the shoulder anatomy, including the critical shoulder angle and glenoid inclination [4-6].

Degenerative partial and complete ruptures of the rotator cuff are common injuries with a steady increase in prevalence with aging [7]. Yamamoto et al [8] reported a total prevalence of rotator cuff tears of 20.7%, with the 0% prevalence in the age group of 20 to 29 years increasing to 50% in those aged 80 to 89 years. The clinical manifestation of degenerative rotator cuff tears varies largely among patients; some have no complaints, and the diagnosis is an incidental finding, while others have severe pain and a limited range of motion that is marginally restored with conservative treatment [9]. In patients with end-stage degenerative rotator cuff lesions, especially with tears of the supraspinatus muscle, this deficit manifests radiologically as a narrowing of the subacromial space [10], presumably caused by the muscular force of the deltoid muscle pulling the humeral head superiorly toward the acromion.

The general population study of Yamamoto et al [8] revealed that 36% of patients with current symptoms had rotator cuff tears, while 17% of asymptomatic participants were also diagnosed with rotator cuff tears. However, to date, possible reasons for this symptomatic discrepancy are poorly understood. Differences in symptomatic and functional limitations may be related to glenohumeral instability in patients with rotator cuff tears. A dysfunctional rotator cuff potentially leads to insufficient joint centering, and tears of the supraspinatus tendon may cause a superior glenohumeral translation. A greater superior translation may affect the pressure around the glenoid cavity and the labrum, possibly leading to pain and impingement and thereby limiting shoulder mobility.

Common kinematic models assume that the glenohumeral joint is a ball-and-socket joint [11], without any consideration of the common clinically observed translation. No conclusive changes in shoulder translation have yet been reported in patients with rotator cuff tears [12-15]. Moreover, it is still unknown how additional handheld weight (comparable to that experienced in situations during daily, occupational, or recreational activities) affects glenohumeral translation in patients with rotator cuff tears. Intact shoulder muscles’ compensation mechanisms for the lacking muscle force of injured muscles are also largely unknown. Such mechanisms may lead to overloading of the uninjured muscles, which can eventually lead to degenerative changes secondary to the original injury.

The current state of research in the field and in our research shows that our proposed framework, applied in a previous pilot study [16] for assessing load-induced glenohumeral translation (liTr), is feasible. Our preliminary clinical analyses have shown that many people aged 45 years and up have degenerative tendon changes despite an absence of symptoms. Clinical observations and results reported in the literature raise the following questions: (1) Is the load-induced increase in deltoid muscle activity with increasing additional load in patients with symptomatic rotator cuff tear greater than that in asymptomatic or healthy joints? (2) Does liTr in the injured joint increase with increasing additional load? (3) Is liTr greater in patients with rotator cuff tears than in age-matched asymptomatic controls and young healthy controls? (4) Is liTr associated with load-induced changes in muscle activation (liMA)? (5) Is there a correlation between the patient’s functional scores and liTr? (6) Is liTr related to muscle morphology, tear size and type, and shoulder anatomy? (7) Are measurements of liTr using motion analysis and single-plane fluoroscopy comparable?

Evidently, there are a variety of factors to consider, including anatomical, morphological, functional, and injury-related factors. Answering these questions will provide information to help clarify the biomechanical limitations of an injured rotator cuff and their impact on treatment, therapy, and daily life. Hence, this study aims to assess the dose-response relationship between liTr and liMA in patients with rotator cuff tears and in age-matched asymptomatic and young healthy participants.

## Methods

### Objectives and Hypotheses

This study will test the overall hypothesis that rotator cuff tears affect glenohumeral translation and that this functional instability depends on the additional load applied, anatomical and morphological variations, and the type and severity of the injury. We propose that greater liMA of the deltoid muscle is associated with greater liTr and that liTr is altered by rotator cuff injury and may be related to patients’ functional scores, muscle cross-sectional area, tear size and type, critical shoulder angle, and glenoid inclination. We also propose that this person-specific relationship is associated with corresponding load-dependent changes in shoulder muscle activity.

Our primary objective is to investigate the dose-response relationship between liMA and liTr in patients with rotator cuff tears and asymptomatic control participants.

Hypothesis 1 is as follows: liMA is positively associated with liTr. Because the shoulder is primarily stabilized by rotator cuff muscles, we expect that the presence of a muscle tear will require additional activity of the remaining intact muscles to stabilize the shoulder. Hence, the dose-response relationship between additional load and relative change in muscle activity with rotator cuff tears will be stronger than in uninjured shoulders.

The secondary objectives are to investigate the in vivo dose-response relationship between additional weight and glenohumeral translation, to understand the biological variation in liTr, the influence of disease pathology on the liTr, the potential compensation by muscle activation and size, and the influence of liTr on patient outcomes. The secondary hypotheses are listed as follows:

- Hypothesis 2.1: liTr is greater in shoulders with rotator cuff tears than in healthy shoulders.
- Hypothesis 2.2: An increase in liTr is part of natural aging, and its variation is related to sex and shoulder anatomy assessed by the critical shoulder angle and the glenoid inclination.
- Hypothesis 2.3: liTr increases with injury severity and depends on injury type.
- Hypothesis 2.4: liMA and side-to-side differences in muscle size (ie, muscle cross-sectional area) increase with the presence of injury and injury severity.
- Hypothesis 2.5: There is a biological variation in liMA and in the compensation effect by muscle activation and muscle size (ie, muscle cross-sectional area).
- Hypothesis 2.6: Large values of liTr are associated with poor functional scores.

In this study, the correlation between liMA and liTr and their relation to patients’ functional scores, muscle activation and size, tear size and type, and shoulder anatomy (critical shoulder angle and glenoid inclination) will be analyzed.

### Study Design and Participants

This study entails cross-sectional, experimental multimodal (clinical, biomechanical, radiological) data collection with multiple conditions and a control group. A total of 25 patients aged 45 to 85 years with unilateral symptomatic rotator cuff tears will be recruited from our clinic. Orthopedic surgeons will inform patients who fulfill the inclusion criteria about the study, and eligible candidates will be contacted. Additionally, 25 asymptomatic controls will be recruited to achieve the same age and sex distribution as in the patient group. Moreover, because of the clinically observed high prevalence of rotator cuff tears in persons without symptoms, we will also recruit 25 sex-matched young asymptomatic controls aged between 20 and 30 years to allow for elucidating the effect of the natural aging process. Detailed inclusion and exclusion criteria for the cohorts are shown in Textbox 1.

**Inclusion and exclusion criteria for this study.**

**Inclusion criteria**

Patients:

- Aged 45 to 85 years (n=25)
- Diagnosed unilateral rotator cuff tear: partial or complete supraspinatus muscle tear, with or without injury to other rotator cuff muscles
- Prior operative treatment of the ipsilateral shoulder or elbow
- Clinical history or symptoms of the contralateral glenohumeral joint
- Range of motion <30° in abduction and flexion

Control participants:

- Two age groups: 20 to 30 years (n=25) and 45 to 85 years (n=25)
- No previous known elbow and shoulder injury or symptoms
- Clinical history of the glenohumeral joint
- Prior conservative or operative treatment of the shoulder or elbow
- Range of motion <90° in abduction and flexion

**Exclusion criteria**

- Inability to provide informed consent
- BMI>35 kg/m^2^
- Neuromuscular disorders affecting upper limb movement
- Additional pathologies influencing the mobility of shoulder joints
- Contraindications for magnetic resonance imaging (eg, neurostimulator and claustrophobia)
- Prior neuromuscular impairment (eg, stroke)
- Diagnosed with active rheumatic disorder
- Other major medical problems
- Pregnancy
- Currently enrolled in another experimental (interventional) protocol

### Experimental Protocol

Each participant will undergo a recruitment and information process before coming to the University Hospital Basel for all planned data collection. Before the assessment, written informed consent will be obtained. Participants will then complete health questionnaires to receive functional scores of the shoulders. Next, fluoroscopic images of a loaded and unloaded 30° shoulder abduction test will be captured, and a 3D motion analysis of the same abduction tests will be performed. Isometric shoulder strength for abduction and internal/external rotation will be assessed with a dynamometer. Finally, magnetic resonance imaging (MRI) images of both shoulders will be taken (Table 1). The estimated total time for each participant is approximately 4 hours. The SPIRIT (Standard Protocol Items: Recommendations for Intervention Trials) checklist, compiled with recommended items to be addressed in a clinical protocol, is provided in Multimedia Appendix 1.

**Table 1:** Schedule of enrollment and assessments.

		Study period	
		Enrollment	Allocation
Time point		Precall	0
**Enrollment**			
	Eligibility screen	✓	
	Informed consent		✓
	Allocation	✓	
**Study groups**			
	Patients		✓
	Control participants (45-85 years)		✓
	Control participants (20-30 years)		✓
**Assessments**			
	Functional scores		✓
	3D motion analysis		✓
	Fluoroscopy		✓
	Dynamometer		✓
	MRI^a^		✓

^a^MRI: magnetic resonance imaging.

#### Functional Scores

Functional scores revealing the patient’s pain, arm range of motion, and ability to perform daily living activities will be assessed using a shortened version of the Disabilities of Arm, Shoulder, and Hand (DASH) questionnaire (QuickDASH) [17] and additional questionnaires, including the Constant Score [18], American Shoulder and Elbow Surgeons Shoulder Score [19], Subjective Shoulder Value [20], and numerical pain rating scale scores. Additionally, participants will complete the EQ-5D-5L [21] questionnaire to assess their general health status.

#### Abduction Test

Participants will be asked to perform a loading/unloading shoulder abduction test until 30°. This test involves abducting the arms to 30° in the scapular plane and then adducting the arms back to the initial (reference) position. All arm movements are performed bilaterally in an upright seated position, with shoulders in the neutral position (hanging arm with thumb toward the front). To control the maximal amplitude of arm abduction, a string is attached to the lower arm and adjusted to the desired maximum position by using a goniometer. Before data collection, participants will be asked to perform exercise movements in the scapular plane. To ensure a comparable speed of movement, verbal commands will be given to the participants.

##### Single-Plane Fluoroscopy

Single-plane fluoroscopic images (Multitom Rax, Siemens Healthineers) will be captured for the following three conditions: without additional weight and in randomized order with 2-kg and 4-kg handheld weights. The rest time between each condition will be at least 30 seconds. Images will be acquired first for the right shoulder independent of the symptomatic side. After a rest period of at least 1 minute, the same tests will be repeated in the same order to obtain images of the left shoulder. The alignment of the scapular plane will be checked by fluoroscopy. Images will be captured with a pulse rate of 10 Hz to minimize radiation exposure. Image dimensions will be calibrated with a reference ball (Ø=25 mm) placed in the field of view.

##### Motion Capture Data

**Figure 1:**
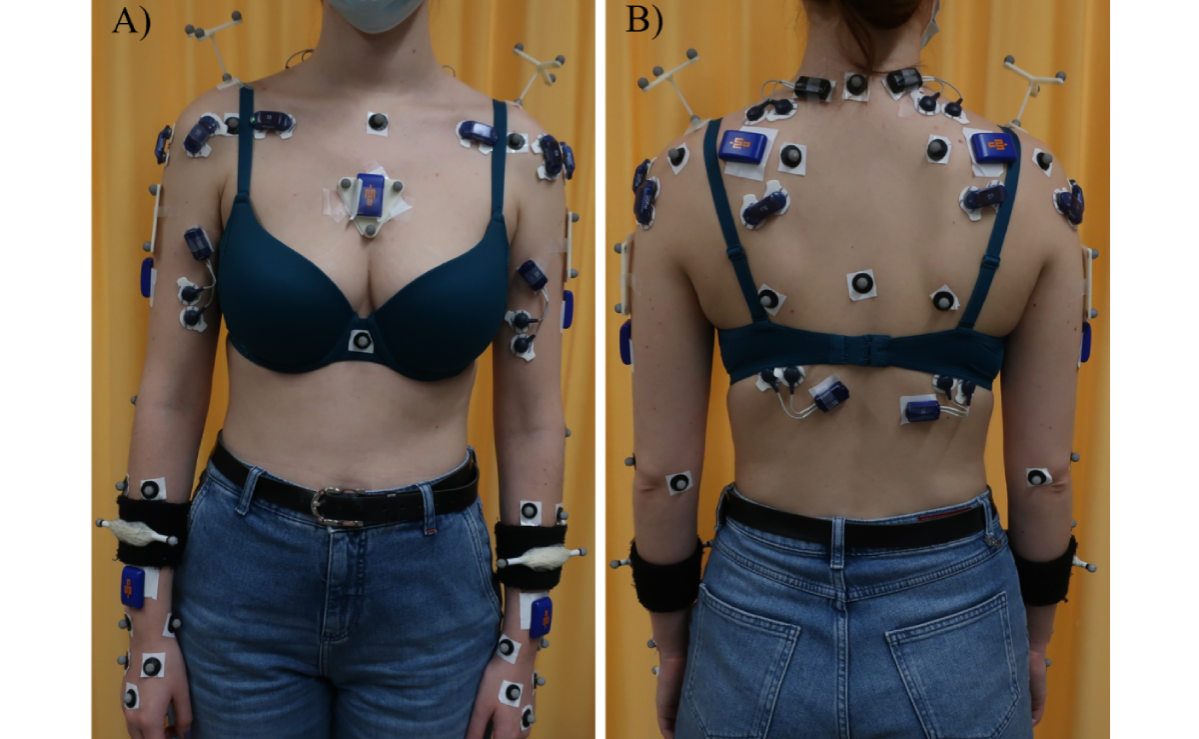
Placement of markers and sensors: (A) anterior and (B) posterior view.

Motion data of this 30° abduction test will be captured with and without additional handheld weights of 1, 2, 3, and 4 kg. The order of the loading conditions will be randomized to minimize fatigue. The task will be interrupted if the participant’s pain exceeds 7 out of 10. Participants will perform 3 trials of each arm movement while kinematic data are collected using a 10-camera Vicon system (Vicon) at a frame rate of 240 Hz. To assess 3D joint angles, retroreflective markers (Figure 1) will be placed on the upper extremity according to the International Society of Biomechanics guidelines [22].

##### Electromyographic Data

Electromyographic data of the infraspinatus, biceps brachii, anterior, middle, and posterior parts of the deltoid, clavicular part of the pectoralis major, latissimus dorsi, and upper trapezius muscles will be recorded during all arm movements. Surface electrodes (Myon AG) will be placed over the corresponding muscles following the guidelines of the SENIAM (Surface ElectroMyoGraphy for the Non-Invasive Assessment of Muscles) project [23] and Criswell [24] (Figure 1). Electromyographic signals will be normalized using the maximal voluntary isometric contraction recorded during the following standard tests (captured during a 5-second trial): empty can, internal rotation 90°, flexion 125°, palm press, and extension [25,26]. Since these tests do not specifically account for the biceps brachii, an additional trial will be captured during maximal contraction against resistance with the elbow flexed at 90°. In the case that a participant cannot reach 90°/125° in flexion or 90° in abduction, these tests will be adapted to the participant’s capabilities by reducing the test angles.

##### Inertial Sensors

Inertial sensor data will also be collected during the loaded and unloaded abduction tests. Additionally, participants will be asked to perform full arm abduction, flexion, and internal and external rotations movements (without additional handheld weight). Inertial sensors (Vicon Blue Trident) will be placed on the participants’ thorax, scapulae, humeri, and forearms [27] (Figure 1). Accelerations and angular velocity will be captured for the various movements.

#### Isometric Shoulder Strength

Shoulder strength will be tested under isometric conditions using a dynamometer (Biodex System 4 Pro, Biodex Medical Systems). Isometric shoulder strength will be assessed for abduction at 10° and 30° in the scapular plane. The participant will be in a seated position with the arm extended in neutral rotation. The protocols comprise 3 repetitions of 5 seconds of pushing and 5 seconds of resting for both abduction angles. Isometric shoulder strength will also be measured for internal and external rotation at neutral rotation in 15° abduction (scapular plane). Participants will be seated with a 90° flexed elbow [28]. The protocol comprises 3 repetitions of 5 seconds of external rotation and 5 seconds of internal rotation with a 5-second rest in between. Both shoulders will be tested in a randomized order. The maximum value of 3 trials will be calculated and recorded as the participant’s maximum isometric strength for each movement.

#### MRI Visualization

MRI will be performed using a 3T scanner (Prisma, Siemens Healthineers) with dedicated shoulder and body array coils. No contrast agent will be administered to the participants. Both shoulders will be scanned. The MRI protocol consists of an axial proton density turbo spin echo (TSE) sequence with fat saturation, a sagittal T1-weighted TSE sequence, a sagittal and a coronal T2-weighted BLADE sequence, and a coronal T1-weighted volumetric interpolated breath-hold examination Dixon sequence (Table 2).

**Table 2:** Characteristics of the MRI^a^ sequences.

MRI sequence	Repetition time/echo time (ms)	Slice thickness (mm)	Field of view (mm)	Matrix
PD^b^ TSE^c^ FS^d^ axial	4500/34	2	140	320 × 320
T1 TSE sagittal	762/11	3	159	384 × 384
T2 BLADE sagittal	4210/72	3.6	160	320 × 320
T2 BLADE coronal	3400/75	3.6	150	320 × 320
T1 VIBE^e^ Dixon coronal	4.04/1.23 (TE^f^1) and 2.46 (TE2)	1.3	238 × 332	184 × 256

^a^MRI: magnetic resonance imaging.

^b^PD: proton density.

^c^TSE: turbo spin echo.

^d^FS: fat saturation.

^e^VIBE: volumetric interpolated breath-hold examination.

^f^TE: echo time.

### Outcome Parameters

The primary and secondary end points are summarized in Textbox 2.

**End points of the experimental protocol.**

**Primary**

- Load-induced glenohumeral translation (liTr) from fluoroscopy
- Load-induced muscle activation of the deltoid muscle

**Secondary**

- liTr from motion capture
- Anatomical parameters
- Injury type
- Functional scores

**Other**

- Age, body height, body mass, and sex of the participant
- Isometric shoulder muscle strength
- Muscle cross-sectional area and fatty infiltration
- Conservative treatment and duration of physiotherapy

#### liTr Measure

**Figure 2:**
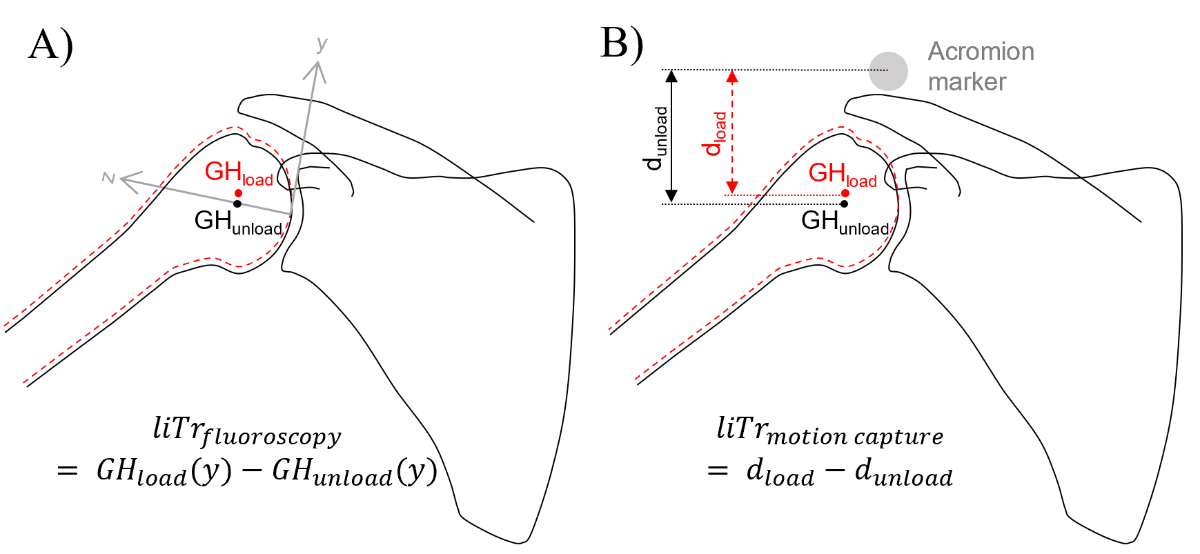
Load-induced glenohumeral translation (liTr). (A) Fluoroscopy-based and (B) motion capture–based measurements. GH: glenohumeral joint center.

Fluoroscopy-based and motion capture–based liTr will be measured (Figure 2).

On the fluoroscopic images, the glenohumeral joint center, humeral shaft, most lateral point of the acromion, and inferior and superior glenoid edges will be registered. The glenohumeral joint center will be determined as the geometric center of a circle comprising the articulating surface of the humeral head [29-32]. Hence, glenohumeral translation during arm abduction and adduction will be measured as the inferior-superior component of a glenoid coordinate system [33] (Figure 2A). The arm abduction angle will be measured as the angle between a line passing through the glenohumeral joint center, the humerus shaft midpoint, and the vertical line.

Glenohumeral translation will also be assessed using instrumented motion analysis. The calibrated scapular and humeral landmarks will be used along with equations outlined by the International Shoulder Group [34] and the functional joint center method to determine the instant glenohumeral joint center within the humerus reference system. The vertical distance from the glenohumeral joint center to the acromion marker will be calculated for a neutral trial and with the arm in 30° shoulder abduction for each loading condition. Motion capture–based liTr will be calculated for each participant as the differences in the distance between the glenohumeral joint center and the acromion under different loading conditions (Figure 2B).

#### liMA Measure

First, an independent component analysis–based filtering will be applied to the electromyographic data to remove electrocardiogram contamination [35]. Then, additional noise artifacts will be removed by a modified bandpass filter of 10 Hz to 450 Hz [36]. For each muscle, liMA will be computed for each loading condition of the abduction test as the root mean square of the electromyographic signal during the loaded arm positions relative to the root mean square of the electromyographic signal during the unloaded arm position [37]. Electromyographic data will be normalized to the maximal voluntary isometric contractions for visual presentation of the electromyographic trajectories.

#### Critical Shoulder Angle

Participants’ critical shoulder angle will be measured on an anterior-posterior double-obliquity fluoroscopy image of the shoulder. This is defined as the angle subtended by a line parallel to the glenoid and a line through the inferior-lateral edge of the glenoid and the inferior-lateral edge of the acromion [5]. The critical shoulder angle has been shown to be reproducible and significantly greater in patients with rotator cuff tears than in the general population [5]. High angles (>35°) have been associated with rotator cuff tears and greater joint instability [38]. Although it can be assumed that there are no side-to-side differences in the critical shoulder angle [39], it will be measured for both shoulders of all the participants (left and right).

#### Glenoid Inclination

The glenohumeral joint is also affected by the orientation of the glenoid; thus, the glenoid inclination can aid our understanding of various shoulder conditions. Indeed, an abnormal glenoid inclination might be associated with rotator cuff tears and superior glenohumeral translation [40]. For each participant, the glenoid inclination will be measured on the initial fluoroscopy image as the angle between a line from the upper to the lower glenoid rim (glenoid plane) and a second line set on the floor of the supraspinous fossa [6,40].

#### Greater Tuberosity Angle

The greater tuberosity angle will be measured on the initial fluoroscopy image as the angle between a line parallel to the humerus diaphysis through the glenohumeral joint center and a line from the upper border of the humeral head to the most superolateral edge of the greater tuberosity [41]. A greater tuberosity angle over 70° has been observed to predict rotator cuff tears [41].

#### Subacromial Space

The subacromial space will be measured on the initial fluoroscopic image as the shortest acromiohumeral distance [42,43]. An acromiohumeral distance between 7 mm and 14 mm is usually considered normal, but below 7 mm, has been associated with rotator cuff tears [43]. A narrow subacromial space might compress the supraspinatus tendon, causing pain [44,45].

#### Muscle Cross-sectional Area

Measurements of the muscle cross-sectional area will be performed on the MRI images using dedicated software (Sectra PACS, Sectra Medical Systems). The cross-sectional area of all rotator cuff muscles will be measured at 2 different positions on parasagittal reformatted images [4]. The cross-sectional area of the deltoid will also be measured on the axial plane at the middle of the glenoid [4].

#### Tear Size, Type, and Location

Information about tear size, type, and location will be retrieved from MRI images and previous shoulder reports or previous radiological shoulder images of the patients, if available. Tear size will be classified into (1) partial or (2) complete supraspinatus tendon tear. Tear type will be classified into supraspinatus tendon tear without injury to other rotator cuff tendons (type A) and with injury to other rotator cuff tendons muscles (type B). Both will be used as indicators for injury severity. Additionally, the value of a combined classification will be investigated. Tear location will be defined as the distance from the anterior margin of the tear at the footprint to the intra-articular portion of the bicep. This will allow us to assess whether most anterior fibers of the supraspinatus tendon are intact [46,47]. Tear location will also be analyzed in comparison to shoulder strength and muscle activity. Partial and full-thickness ruptures will be classified according to the classifications of Ellmann [48] and Patte [49], respectively, allowing us to evaluate how advanced the rotator cuff tear is.

### Statistics and Determination of Sample Size

All study-related data will be entered into and stored using a web-based Research Electronic Data Capture (REDCap; Vanderbilt University) system [50,51]. All statistical analyses will be performed in R statistical software [52]. Overall, 25 patients with 1 symptomatic shoulder with a rotator cuff tear, 25 age- and sex-matched asymptomatic control participants, and 25 sex-matched young control participants will participate in this study. This means that a total of 150 shoulders will enter the analysis. Comparisons between the fluoroscopy-based liTr and motion capture–based liTr will be made. Reproducibility and agreement will be assessed by the mean and standard deviation of differences and Bland-Altman plots. Precision in estimating the liTr will be assessed by average relative standard errors and *R*^2^ values from a global model. Potential deviations from a linear relationship will be assessed by random effects models for a quadratic term. The influence of covariates on the liTr and the biological variation of liTr will be assessed by mixed models applied to the original glenohumeral translation measurements with the liTr as a random effect. Dependence between the shoulders will be considered by bivariate random effects.

#### Participant Characteristics

Descriptive statistics will be used to describe the characteristics of the participants in each group. Mean and standard deviation of age, sex, body height, body mass, BMI, conservative treatment (if applicable), time since injury, and duration of physiotherapy will be calculated to describe the participants.

#### Sample Size Calculation

**Figure 3:**
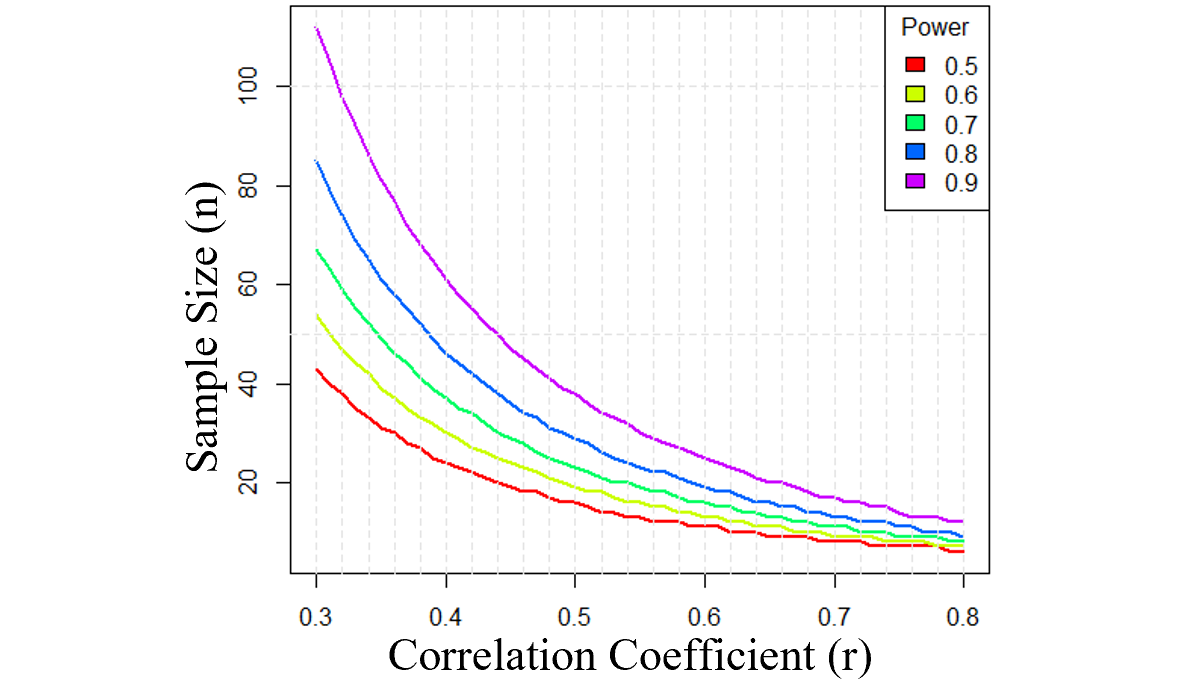
Sample size estimation for different scenarios for power and correlation.

From a pilot study data consisting of 18 participants (8 patients and 10 asymptomatic controls), we found a correlation of 0.37 between the liTr and liMa for the treatment group and an overall correlation of 0.63 for all individuals included in the study [16]. Using these findings, we considered different correlation scenarios between liTr and liMa for a different power of analysis (Figure 3). For a 5% significance level, 90% power, and a correlation of 0.37, we need 76 shoulders. Considering a possible dropout rate of 5%, we need 80 shoulders for the first hypothesis in this study. We also powered the analysis for the secondary hypotheses and used the repeatability investigation, which suggested a measurement error with a standard deviation of 0.16 mm for the sphere method when assessing the liTr by comparing a load of 3 kg with no load. Moreover, the pilot study revealed a population standard deviation of 1.09 mm for the liTr, suggesting a substantial biological variation in the liTr. Thus, it should be possible to estimate this variation and assess the effect of covariates. Based on our clinical findings, we expect to observe incidental findings of rotator cuff tears in two-thirds of asymptomatic shoulders in participants aged 45 years or older. Therefore, we expect to have 75 shoulders with rotator cuff tears. Within the 75 shoulders with rotator cuff tears, it will be possible to detect a difference of 0.8 mm in liTr with a power of 90%.

We adopted this sample size calculation post hoc after the aforementioned clinically relevant finding. We kept the recruitment of 75 participants but adapted the study post hoc to 3 groups with 25 participants in each. For any significant findings, this post hoc adaptation will be further investigated in the analysis stage by power calculation to assess potential limitations for the main analysis and reporting of results to avoid relying on findings by chance.

### Ethics Approval

This study protocol was approved by the Ethics Committee Northwest Switzerland (2021-00182; Multimedia Appendix 2) and registered on ClinicalTrials.gov (NCT04819724). Prior to participation, written informed consent will be obtained from all participants. Participants can withdraw from the study at any time. This study is conducted in accordance with the Declaration of Helsinki, the principles of Good Clinical Practice, the Human Research Act, and the Human Research Ordinance, along with other locally relevant regulations.

### Safety Considerations

Fluoroscopy data will be used in this study to measure the glenohumeral translation. Fluoroscopy is a low-dose x-ray application routinely used in clinical practice. The estimated effective dose will be maximally 0.01 mSv. A standard x-ray of the chest, which is considered to have minimal radiation exposure, has an effective dose of 0.1 mSv and is comparable to exposure to 10 days of natural background x-ray volume [53]. To limit radiation exposure, fluoroscopy images will only be taken for 0-, 2-, and 4-kg handheld weights during the 30° abduction test in the scapular plane. Therefore, 1- and 3-kg handheld weights will not be included in the fluoroscopy-based liTr measurements.

## Results

Recruitment and data collection began in May 2021, and they will last until the recruitment target is achieved. Data collection is expected to be completed by the end of 2022. As of November 2022, data processing and analysis are in progress, and the first results are expected to be submitted for publication in 2023.

## Discussion

This experimental protocol will allow for a comprehensive analysis of clinical, functional, and biomechanical data. Furthermore, using MRI, asymptomatic rotator cuff tears will be revealed, aiding our understanding of the effect of rotator cuff tears on glenohumeral motion, especially in asymptomatic shoulders. We expect that rotator cuff tears will have a greater glenohumeral translation and that this will depend on the additional load applied and the anatomical parameters. In addition, a greater liMA of the deltoid muscle is expected with greater liTr. These outcomes might also be associated with muscle cross-sectional area, tear size, and type.

With our infrastructure, electromyographic/motion capture data and fluoroscopy images cannot be acquired simultaneously. Hence, we cannot completely rule out differences in the execution of the abduction tests. However, prior to data collection, exercises will be practiced, and verbal commands will be given to ensure comparable movements.

Glenohumeral translation is an important surrogate for shoulder instability that represents not only functional limitations but also a risk factor for the development of joint degeneration, including osteoarthritis. The results of this study will provide the first evidence of a dose-response relationship between additional weight and glenohumeral translation in patients with rotator cuff tears. Confirming this dose-response relationship and its impact on functional scores and modulating effects by tear type and size, muscle cross-sectional area, critical shoulder angle, and glenoid inclination will elucidate the importance of limiting additional weight during daily activity in this population. Revealing the effects of additional load on muscle activity of the intact rotator cuff tendon in the injured joint will provide evidence of potential overload in these muscles that may lead to secondary damage in other tissues in the injured joint. This study can be considered a proof of concept of a potential diagnostic test (loading shoulder abduction test) for glenohumeral translation. The recommendation based on our results will directly impact activity limitations in patients with rotator cuff tears and influence treatment choice and rehabilitation regimens. The results of this study will be presented at national and international conferences and published in peer-reviewed open-access journals.

## Appendix

Multimedia Appendix 1 SPIRIT (Standard Protocol Items: Recommendations for Intervention Trials) checklist.

Multimedia Appendix 2 Ethics approval.

Multimedia Appendix 3 External funder confirmation.

Multimedia Appendix 4 External peer-review report by the Swiss National Science Foundation.

## Abbreviations

DASH: Disabilities of Arm, Shoulder, and Hand
liMA: load-induced changes in muscle activation
liTr: load-induced glenohumeral translation
MRI: magnetic resonance imaging
REDCap: Research Electronic Data Capture
SENIAM: Surface ElectroMyoGraphy for the Non-Invasive Assessment of Muscles
SPIRIT: Standard Protocol Items: Recommendations for Intervention Trials
TSE: turbo spin echo
