# Supplementary material for: Load-Induced Glenohumeral Translation After Rotator Cuff Tears: Protocol for an In Vivo Study
Source: JMIR Res Protoc. 2022 Dec 23;11(12):e43769. doi: 10.2196/43769 (PMC9823567; doi:10.2196/43769)
Supplement: Multimedia Appendix 2 [file resprot_v11i12e43769_app2.pdf]

EKNZ

Ethikkommission
Nordwest- und
Zentralschweiz

Präsident
Prof. Christoph Beglinger
Vizepräsidenten
Dr. Angela Frotzler
Dr. Marco Schärer

To whom it may concern

Basel, August 11th, 2022 / NJ

Confirmation of ethic approval for the project "liTrans - Influence of additional weight carrying on load-induced changes in glenohumeral translation in patients with rotator cuff tear – a translational approach
" – BASEC ID 2021-00182

Dear Sir, dear Madam,

We hereby confirm that the project "liTrans - Influence of additional weight carrying on load-induced changes in glenohumeral translation in patients with rotator cuff tear – a translational approach", led by Prof. Dr. Annegret Mündermann, has been approved by the Ethics Committee Northwest and Central Switzerland (EKNZ) on March 8th, 2021.

Yours sincerely,

i.V. [illegible]

Prof. Dr. Christoph Beglinger, MD
President of the Ethics Committee
Northwest and Central Switzerland / EKNZ

Geschäftsführerin Irene Oberli | Hebelstrasse 53 | 4056 Basel | Tel 061 268 13 50 | eknz@bs.ch | www.eknz.ch
