# Supplementary material for: Load-Induced Glenohumeral Translation After Rotator Cuff Tears: Protocol for an In Vivo Study
Source: JMIR Res Protoc. 2022 Dec 23;11(12):e43769. doi: 10.2196/43769 (PMC9823567; doi:10.2196/43769)
Supplement: Multimedia Appendix 3 [file resprot_v11i12e43769_app3.pdf]

Swiss National
Science Foundation

Unit Projects Life Sciences
Wildhainweg 3, P.O. Box
3001 Berne

+41 31 308 22 22
Projects.ls@snf.ch
www.snsf.ch

To whom it may concern

Berne, 17 June 2022

**320030_189082**

The Swiss National Science Foundation SNSF is the main public funding agency for academic research in Switzerland.

We herewith confirm that the study protocol for the project 320030_189082 "Influence of additional weight carrying on load-induced changes in glenohumeral translation in patients with rotator cuff tear - a translational approach” was selected for funding after independent peer review. The SNSF has no self-interest in the study outcomes.

Yours sincerely

Christoph Meier, PhD
Head of Unit Projects Life Sciences
