# Supplementary material for: Load-Induced Glenohumeral Translation After Rotator Cuff Tears: Protocol for an In Vivo Study
Source: JMIR Res Protoc. 2022 Dec 23;11(12):e43769. doi: 10.2196/43769 (PMC9823567; doi:10.2196/43769)
Supplement: Multimedia Appendix 4 [file resprot_v11i12e43769_app4.pdf]

Review: 1

Application data

Applicant(s)

Mündermann, Annegret
Baumgartner, Daniel / Müller, Marc Andreas

Influence of additional weight carrying on load-induced changes in glenohumeral translation in patients with rotator cuff tear - a translational approach

Project funding in biology and medicine (division III)

Detailed evaluation

Applicants' scientific track record and expertise

See overall comments.

Scientific relevance, originality and topicality

See overall comments.

Broader impact (forms part of the assessment of scientific relevance, originality and topicality)

See overall comments.

Suitability of methods and feasibility

See overall comments.

Comment

Overall Comments:
Overall, I think this is an excellent proposal for several important reasons including the bio mechanical issues that occur following a rotator cuff tear. Several comments have come about. Please include consideration of proposing shoulder biomechanics at the beginning of a tear. There should also be research on scapular kinematics. The reason for this comes from the North American literature on scapular biomechanics and shoulder disease. I know that there was mention but perhaps more details. The reason this is suggested as it speaks to the value of physiotherapy and managing the majority of these cases with just PT. This should certainly be a consideration.
Given the area of expertise and that there is an increase incidence of scapular dyskinesia following cuff tears, this would be a critical piece.
This group of studies will thoroughly provide foundational knowledge on biomechanics following pathology. It will add to the existing body of literature.

Note on the evaluation procedure

The evaluation bodies of the SNSF strive to reach a balanced overall assessment of each proposal. External reviews play an important role in this. Reviewers generally review only one proposal. The evaluation bodies of the SNSF, however, must compare and rate the quality of all proposals submitted by a given deadline. The opinions expressed in external reviews are generally positive, or they may occasionally include critical remarks that are largely irrelevant to the assessment conducted by the evaluation body. Therefore, the final decision taken by the SNSF evaluation bodies need not necessarily reflect the content of external reviews.

Review: 2

Application data

Applicant(s)

Mündermann, Annegret

Baumgartner, Daniel / Müller, Marc Andreas

Influence of additional weight carrying on load-induced changes in glenohumeral translation in patients with rotator cuff tear - a translational approach

Project funding in biology and medicine (division III)

Detailed evaluation

Applicants' scientific track record and expertise

All the applicants are dedicated clinician scientists, with an excellent records of generating basic sciences ideas, testing them in a laboratory setting, and at least trying to translate them into clinical practice.

Scientific relevance, originality and topicality

The ideas behind the proposal are sound, and original. The scientific relevance is well discussed, and I have no doubts that the expected results will be scientifically valid. The rate of rotator cuff injury is high, and the prevalence of such ailment is likely to increase given the increased life expectancy. Problems in the rotator cuff are also prevalent in athletes, and therefore the project has implication for this patients population as well.

Broader impact (forms part of the assessment of scientific relevance, originality and topicality)

Please see above.

Suitability of methods and feasibility

The laboratory work is well outlined, and the previous work in this field performed by the authors in their own laboratories brings me to consider that what is proposed extends their previous endeavours.

The time line is a bit tight, but I suspect it reflects the fact that the applicants know that, given their past experience, they can satisfy it.

The clinical measurements in humans are topical, and my only concern is that 75 patients and 25 controls may not be enough to provide normative data.

Comment

Overall, I would classify this project as excellent.
Strengths: Excellent track record of the applicants, dedicated laboratories, well planned out experiments.
Weaknesses: Tight time line, concerns about production of normative data from a relatively small populace.

Note on the evaluation procedure

The evaluation bodies of the SNSF strive to reach a balanced overall assessment of each proposal. External reviews play an important role in this. Reviewers generally review only one proposal. The evaluation bodies of the SNSF, however, must compare and rate the quality of all proposals submitted by a given deadline. The opinions expressed in external reviews are generally positive, or they may occasionally include critical remarks that are largely irrelevant to the assessment conducted by the evaluation body. Therefore, the final decision taken by the SNSF evaluation bodies need not necessarily reflect the content of external reviews.
